# Modelling maternal cardiovascular adaptation to pregnancy: a scoping review

**DOI:** 10.1186/s12884-025-08270-z

**Published:** 2025-11-24

**Authors:** Kathryn Hunt, Ioannis Polydoros, Raoul van Loon, Rosemary C. Townsend, Rebecca M. Reynolds

**Affiliations:** 1 Centre for Cardiovascular Science, University of Edinburgh, Edinburgh, UK; 2 Biomedical Engineering Simulation and Testing Lab, Department of Biomedical Engineering, Swansea University, Swansea, UK; 3 Medical Research Council Centre for Reproductive Health, University of Edinburgh, Edinburgh, UK

**Keywords:** Pregnancy, Cardiovascular dysfunction, Computational modelling, Digital twins, Pre-eclampsia, Fetal growth restriction

## Abstract

**Background:**

The maternal cardiovascular system undergoes a profound transformation during pregnancy. Disordered cardiovascular adaptation is associated with pre-eclampsia and fetal growth restriction; however, these complications remain challenging to predict and manage. Computational modelling presents an opportunity to study the maternal circulation with unprecedented flexibility.

We conducted a scoping review to assess the potential utility of computational models for studying maternal cardiovascular adaptation to pregnancy and providing insights into disease. We aimed to identify research gaps and barriers to translating this emerging field into clinical practice.

**Methods:**

Medline, Embase, and Web of Science Core Collection were searched from 01/01/2013 to 01/09/2025, for articles related to computational haemodynamic models of pregnancy. English-language studies describing models of the maternal circulation in human pregnancy were identified. Information on system modelled, study design, participant or clinical specimen details, and key findings were extracted from original research studies and presented in a narrative synthesis.

**Results:**

Of 662 citations, 37 met inclusion criteria, comprising 28 original research studies, two conference abstracts, and seven reviews. Amongst the original research studies, 27 were basic experimental papers and one was a retrospective cohort study. Nine experimental articles incorporated data from pregnant participants or clinical specimens into personalised models, with sample sizes between one and 21. Four of these included participants or specimens affected by hypertensive disorders of pregnancy or fetal growth restriction. The retrospective cohort study employed haemodynamic modelling to investigate blood loss in 480 patients with abnormally invasive placentas.

Modelling approaches described included those simulating the entire maternal circulation, maternal cardiac remodelling, maternal renal autoregulation, the maternal pelvic circulation in isolation, uterine vascular adaptation, spiral artery remodelling, and blood flows within the placental intervillous space.

**Conclusions:**

Computational modelling could represent a powerful tool to advance understanding of maternal cardiovascular adaptation to pregnancy and guide future approaches to risk stratification, diagnosis, and management of pregnancy complications. Realising this promise will require cardiovascular models to be parameterised with scalable metrics, validated in large pregnancy cohorts, developed alongside robust computational processing pipelines, and integrated into existing clinical workflows.

**Protocol registration:**

The review protocol was registered prospectively with the Open Science Framework (osf.io/v3968).

**Supplementary Information:**

The online version contains supplementary material available at 10.1186/s12884-025-08270-z.

## Background

### The maternal cardiovascular system in normal and disordered pregnancy

During healthy pregnancy, dramatic changes occur in the maternal cardiovascular system to facilitate an expansion of circulating volume and redistribution of blood flow towards the uterus. Cardiac output increases by up to 45% between 5 and 25 weeks’ gestation, whilst generalised vasodilation mediates a fall in total peripheral resistance [1]. This process of cardiovascular transformation occurs differently in pre-eclamptic pregnancies, suggesting a maladaptive response to gestation in affected women [2].

Pre-eclampsia is a heterogenous condition; early onset-forms are often associated with fetal growth restriction and display different trends in cardiovascular parameters to late-onset disease, where fetal growth is usually appropriate [3, 4]. Disordered placental vascular development is common to both pre-eclampsia and fetal growth restriction [5, 6]. However, it is not clear whether characteristically abnormal patterns of spiral artery remodelling represent a primary placental pathology, or are secondary to inappropriate flow conditions in the maternal uterine vasculature due to insufficient cardiovascular adjustment to early pregnancy [7].

Beyond the affected pregnancy, women who are diagnosed with pre-eclampsia have increased risk of recurrent disease in subsequent pregnancies and persistently elevated lifetime cardiovascular risk profiles, with higher rates of hypertension, ischaemic heart disease, and related mortality, compared to their counterparts with uncomplicated pregnancies [8–10]. A pertinent unanswered question is whether this is because pregnancy “unmasks” an underlying cardiovascular vulnerability which manifests as pre-eclampsia, or whether instead the widespread endothelial dysfunction seen in the disease results in vascular remodelling and confers cumulative cardiovascular susceptibility with each affected pregnancy [11–13].

The gaps in our understanding of the underlying pathophysiology of pre-eclampsia severely hamper the ability of clinicians to predict this common pregnancy complication and mitigate its most devastating effects, which can include stillbirth, maternal death, and the acute and chronic sequelae of iatrogenic preterm birth [14]. We propose that novel, computational approaches may have a role in untangling the thorny and persisting dilemmas posed by this enigmatic disease.

### The opportunity presented by computational modelling

Studying cardiovascular adaptation to pregnancy is challenging. Invasive measurement techniques and exposure to ionising radiation, unless justified by clinical necessity, are unethical in pregnant participants. Assessment of vascular anatomy and measurement of blood flow are therefore restricted to ultrasound or magnetic resonance imaging (MRI)-based approaches, or to ex vivo examination of placental morphology and histology following birth.

Preclinical animal models of pre-eclampsia have been used historically to probe genetic, molecular, and physiological mechanisms underlying abnormal maternal cardiovascular responses [15]. However, such models have limited applicability to human pregnancy due to significant difference in scale, placental anatomy, trophoblast invasion and uterine vascular remodelling between species [16].

Where studies of pregnant human participants are attempted, these generally rely upon clinic-based measurements or imaging assessments, providing snapshots of cardiovascular status at a handful of timepoints. These may not fully reflect the dynamic process of cardiovascular adaptation, particularly given that maternal haemodynamic status fluctuates considerably in response to changes in volume status, circadian variation, positional changes, external stressors, and antihypertensive therapies [17, 18].

Advances in bioengineering, computational fluid dynamics, and machine learning are enabling complex physiological systems and haemodynamic processes to be modelled with ever-increasing precision, including within obstetric populations [19–21]. This emerging field presents exciting opportunities to study cardiovascular adaptation to pregnancy at greater resolutions than has previously been feasible in humans. Modern mobile-sensing “wearable” technologies could extend the reach of these computational models, enriching them with patient-specific data and providing insights into dynamic evolution and temporal variations within the maternal circulation.

In this review, we have assessed the potential utility of computational models for furthering our understanding of maternal cardiovascular adaptation to pregnancy, by summarising existing attempts to simulate the pregnant circulation in health and disease. These models may represent an important first step towards a future where personalised medicine, combined with biomedical engineering approaches, will enable nuanced and precise risk stratification, monitoring, and intervention for pregnancy complications. We discuss new insights that computational models have already revealed into the pathophysiology of pre-eclampsia and fetal growth restriction, and identify hurdles which must be overcome in order to realise their clinical potential.

## Methods

This scoping review was conducted using the framework set out in the Joanna Briggs Institute (JBI), and is reported in accordance with the Preferred Reporting Items for Systematic Reviews and Meta-Analyses – Scoping Review (PRISMA-ScR) checklist [22, 23]. The protocol was registered prospectively on the Open Science Framework (OSF) website, using the JBI protocol template (osf.io/v3968).

### Search strategy

Our search strategy combined keywords and subject headings for mathematical or computational models, haemodynamics, vasculature, and pregnancy, using the selection criteria below (see Appendix S1). Searches were conducted in Medline (Ovid), Embase (Ovid), and Web of Science Core Collection from 1 st January 2013 to 1 st September 2025, with no language restriction and no limit on study design. References of retained articles were reviewed for studies not captured in the search, and further studies were added manually following discussion with expert colleagues.

### Selection criteria

We identified English-language articles of any study design, including original research papers, conference abstracts, higher degree dissertations, reviews, and opinion pieces, which described the development or use of mathematic or computational models to simulate blood flows or vascular function in any part of the cardiovascular system during human pregnancy. Studies which included solely models in non-human species were excluded.

### Study selection

Citations were downloaded and screened using Covidence software [24]. Two independent reviewers (KH and IP) screened titles and abstracts of citations to ensure eligibility. Retained citations were then sorted into articles pertaining to the fetal circulation, placental villous circulation, and maternal circulation (down to the level of maternal blood flows within the placental intervillous space). As the focus of this scoping review is the maternal cardiovascular system, full-text papers from the latter category were reviewed by both reviewers before final decisions were made regarding inclusion. Disagreements were resolved through discussion, or recourse to a third reviewer (RL) if necessary.

Where citations were conference abstracts or higher degree dissertations, the reviewers made efforts to identify whether the study described had been published in a peer-reviewed publication. Where citations were review articles or opinion pieces, the reviewers identified any original research studies referenced (which were not already captured by our search) and assessed their eligibility for inclusion.

### Data collection

Data were extracted from included original research studies by one reviewer (KH), using a standard, piloted form. Extracted data were publication details, system modelled, study design and scope, details of human participants or clinical samples, and key findings relevant to the review topic.

### Data synthesis

A narrative synthesis is used to summarise current efforts to build computational models of maternal cardiovascular adaptation to pregnancy.

## Results

### Study selection

**Fig. 1:**
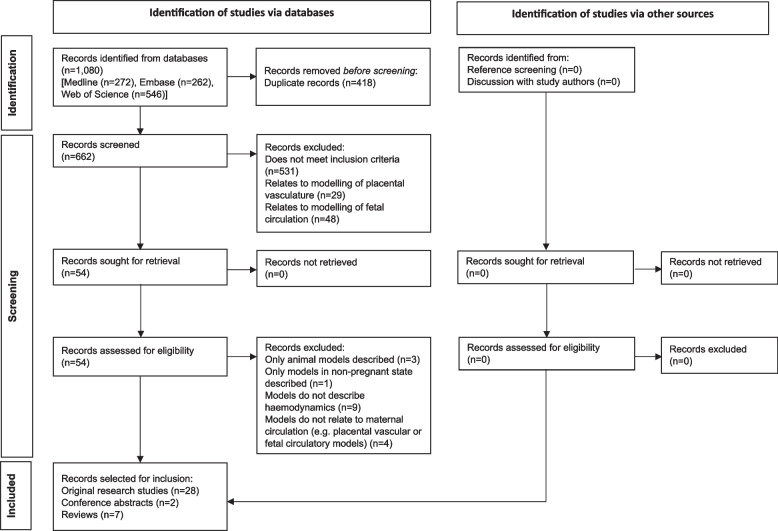
PRISMA flow diagram of article selection

The PRISMA flow diagram is provided in Fig. 1. Following de-duplication, 662 citations from databases were screened. 37 articles were selected that met inclusion criteria and pertained to the maternal cardiovascular system. These comprised 28 original research studies, two conference abstracts and seven reviews [20, 25–60]. Of note, the work detailed in both conference abstracts was subsequently published, and the resultant papers are amongst the included original research studies.

### Study characteristics

Of the original research studies identified, 27 were basic experimental papers and one was a retrospective cohort study. Six of the experimental articles incorporated data from pregnant participants into computational models (sample sizes 2–21), and four incorporated data from histological examination or imaging of ex vivo placental specimens (sample sizes 1–6) [26, 27, 30, 31, 36, 45, 47, 48, 50]. Four experimental papers used data from participants or specimens affected by hypertensive disorders of pregnancy or fetal growth restriction to generate models [27, 31, 36, 47].

In contrast, the retrospective cohort study involved 480 pregnant subjects with abnormally invasive placentas [35].

**Fig. 2:**
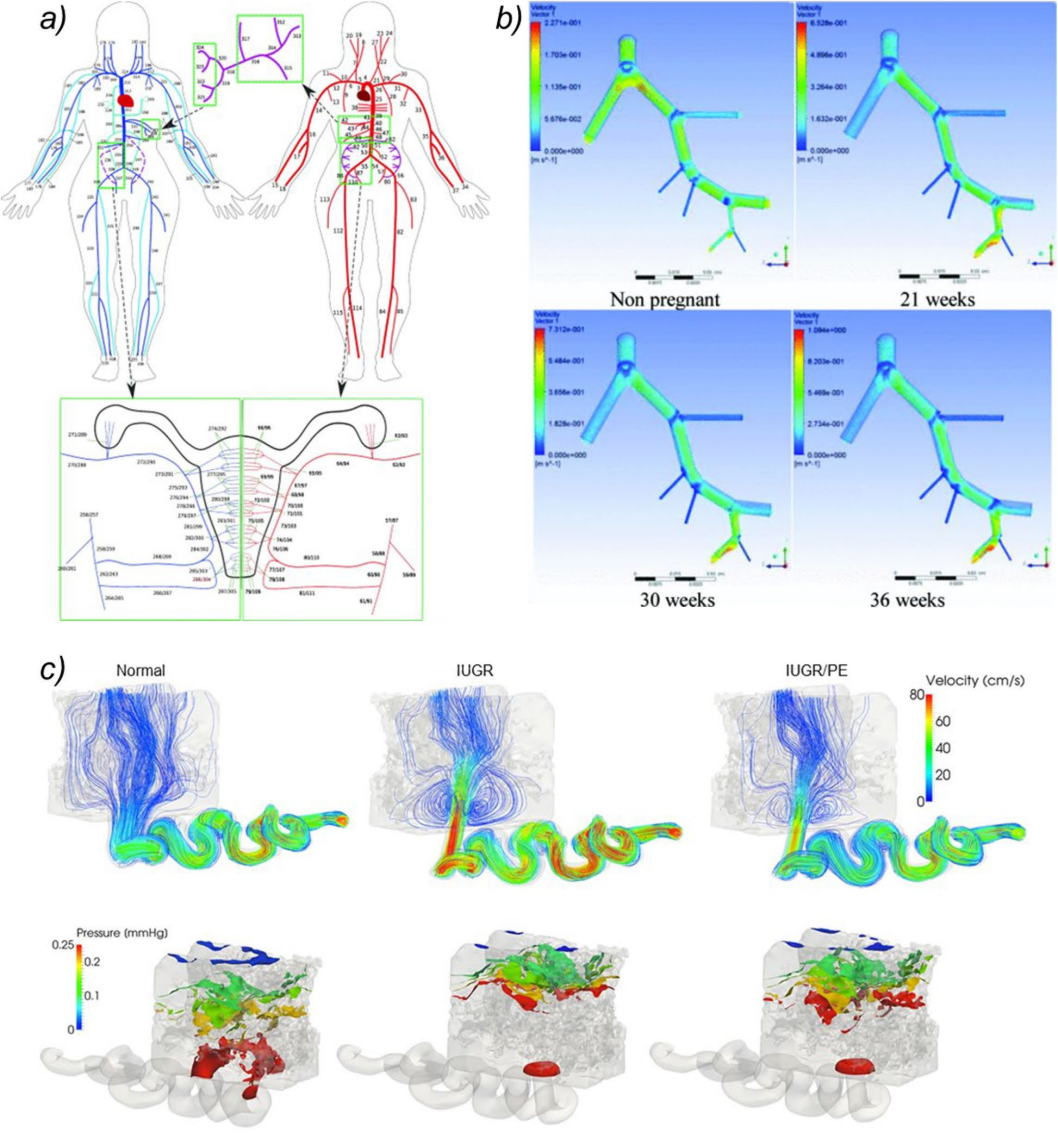
Computational Models of Pregnancy Across Different Biological Scales. (**a)** A closed-loop network model of the pregnant maternal circulation including systemic arteries (red), systemic veins (blue), and hepatic and utero-ovarian vessels (purple) (reproduced from Ref. [26]) (**b**) Three-dimensional fluid dynamics simulations of blood flow within the pelvic arterial tree, from the common iliac to the uterine artery, in the non-pregnant state and at different gestational time points (reproduced from Ref. [34]) (**c**) Simulated blood velocities and pressures, at the maximum systolic flow of a maternal heart cycle, within a spiral artery and proximal intervillous space in normal pregnancy and pregnancies affected by fetal growth restriction alone (IUGR), and fetal growth restriction and pre-eclampsia (IUGR/PET) (reproduced from Ref. [47]) Figures (**a**) and (**c**) reproduced under CC BY 4.0 (https://creativecommons.org/licenses/by/4.0/). Figure (**b**) reproduced with permission from the copyright holder

The scope of modelling approaches reported across studies included those simulating the entire maternal circulation, maternal cardiac remodelling, maternal renal autoregulation, the maternal pelvic circulation in isolation, uterine vascular adaptation, spiral artery remodelling, and maternal blood flows within the placental intervillous space. Examples of models simulating maternal vascular physiology at different biological scales are shown in Fig. 2.

Characteristics and findings of the original research studies are summarised in Table 1. The remainder of this review describes and discusses these studies, in the context of computational modelling approaches to pregnancy as an emerging field.

**Table 1:** Characteristics and findings of included original research studies

Study	System modelled	Objective and scope of study	Human participants or clinical samples	Key findings
Corsini et al. 2017 [25]	Entire maternal circulation	To develop a mathematical model of the pregnant circulation which reflects haemodynamic trends in each trimester	None	Model-predicted cardiovascular parameters and uterine artery waveforms aligned with literature values for healthy pregnancies
Carson et al. 2019 [26]	Entire maternal circulation	To incorporate the utero-ovarian communicating arteries into a model of the pregnant circulation	*n* = 2 healthy pregnant participants	Model-predicted cardiovascular parameters and uterine artery waveforms aligned with clinical measurements and typical literature values, respectively, for healthy pregnancies
Carson et al. 2021 [27]	Entire maternal circulation	To validate an existing model of the pregnant circulation with patient-specific measurements	*n* = 4 pregnant participants in the second trimester - 2 had early-onset placental disease - 2 had chronic hypertension but otherwise uncomplicated pregnancies	Model-estimated uterine artery waveforms had similar characteristics to measured waveforms Model-predicted areas of main systemic arteries were greater for participants with placental disease compared to those without placental disease
Gleason et al. 2022 [28]	Entire maternal circulation	To incorporate vascular growth and remodelling into a model of the pregnant circulation	None	Model-predicted cardiovascular parameters and uterine artery waveforms aligned with literature values for healthy pregnancies, from conception to 40 weeks
Sedaghati et al. 2023 [29]	Entire maternal circulation	To establish whether a model of the pregnant circulation which incorporates vascular growth and remodelling can reflect haemodynamic trends in early and late forms of pre-eclampsia	None	Model-predicted cardiovascular parameters aligned with literature values Model predicted diameter of the ascending aorta increased in uncomplicated pregnancy and late pre-eclampsia, and remained constant in early pre-eclampsia Model-predicted aortic stiffness increased throughout gestation to a greater degree in pre-eclampsia than in uncomplicated pregnancy
Ormesher et al. 2023 [30]	Entire maternal circulation	To use a model of the pregnant circulation to investigate the effects of maternal prone position	*n* = 21 healthy pregnant participants in the third trimester	Model-predicted falls in cardiac output during prone positioning corresponded to measured trends, suggesting vena cava compression could account for this effect Observed blood pressure rose in prone position, in contrast to the decrease predicted by the computational model—the authors suggest this may be a result of autoregulatory mechanisms or due to gravity causing discordance between aortic blood pressure and pressure measured by a brachial cuff
Popp et al. 2024 [31]	Entire maternal circulation	To use modelling of the pregnant circulation to identify novel haemodynamic biomarkers which can distinguish pregnancies affected by early-onset placental disease from unaffected pregnancies	*n* = 21 pregnant participants in the second trimester - 9 with early-onset placental disease (pre-eclampsia or fetal growth restriction) - 12 without early-onset placental disease (including uncomplicated pregnancies, late-onset pre-eclampsia, and late-onset fetal growth restriction)	Biomarkers based on model-predicted radial or arcuate artery blood flow properties had a higher classification ability for early-onset placental disease in the study dataset than measured blood pressure or uterine artery pulsatility or resistive indices
Comunale et al. 2021 [32]	Maternal cardiac remodelling	To incorporate a cardiac remodelling algorithm into a circulatory model of pregnancy	None	A realistic pattern of pregnancy-induced heart growth could be simulated by a model constrained by maintaining homeostasis of cardiac myofiber stress and wall shear stress
van Ochten et al. 2022 [33]	Maternal renal autoregulation	To incorporate renal autoregulation into a circulatory model of pregnancy	None	When the model was parameterised using human literature values for 8 weeks gestation, predicted changes in renal blood flow in response to blood pressure aligned with renal autoregulatory responses (as seen in rat studies)
Serrano et al. 2019 [34]	Maternal intrapelvic arterial circulation	To develop a 3D model of the pregnant maternal pelvic arterial tree in the second and third trimester	None	Model-predicted blood flows and velocities followed a similar pattern to literature values
Li et al. 2021 [35]	Maternal intrapelvic circulation	To use a circulatory model to simulate observed blood loss at delivery in cases treated with internal iliac artery balloon occlusion (IIABO) compared with controls, in a retrospective analysis of cases with pernicious placenta praevia	*n* = 480 pregnant participants with pernicious placenta praevia - 288 treated with IIABO - 192 controls	Model simulations revealed that observed blood losses could be explained by the presence of collateral circulation and reversed flow in uterine venules
Kissas et al. 2022 [36]	Maternal intrapelvic arterial circulation	To develop a computational model of the pelvic arterial tree which can predict patient-specific blood flow and pressure waveforms throughout the network from MRI measurements	*n* = 2 pregnant participants in the second trimester - 1 developed pregnancy induced hypertension in late pregnancy - 1 with uncomplicated pregnancy	Differences were observed in model-predicted vessel resistances and velocity and pressure waveforms between participants (e.g. higher pressures and lower velocities in pre-hypertensive subject) There were substantial discrepancies between MRI-measured and model-predicted results
Clark et al. 2018 [37]	Maternal uterine circulation	To develop a model of the uteroplacental circulation which can predict uterine artery waveforms in the second trimester and post-delivery	None	Models with narrow calibre radial arteries generated high resistance uterine artery waveform patterns Blood shunting through arteriovenous anastomoses was necessary to explain low resistance uterine artery waveforms post-delivery
Allerkamp et al. 2023 [38]	Maternal uterine circulation	To incorporate trophoblast plugging and remodelling of spiral arteries into a computational model, to predict shear stress in upstream radial arteries in early pregnancy (for use in cellular shear stress assays)	None	Reduced radial artery radius, premature removal of spiral artery trophoblast plugs, and increased channel size in trophoblast plugs all increased shear stress in radial arteries In cellular shear stress assays, increasing physiological levels of shear stress reduced the effect of paracrine factors on endothelial nitric oxide production
Felias et al. 2025 [39]	Maternal uterine circulation	To develop a 3D model of the uterine artery and combine this with data from Doppler ultrasound studies, to predict haemodynamic properties including wall shear stress and oscillatory shear index	Doppler ultrasound data from > 200 pregnant women in the second and third trimesters were used to define mean values of peak systolic and end diastolic blood flow for use in the model, but not to generate individual-specific simulations	Model simulations suggested that uterine artery blood flow in the second trimester is laminar and stable, with minimal pressure fluctuations and low velocity variation
James et al. 2018 [40]	Maternal spiral artery	To develop a model of a spiral artery “plugging” by extravillous trophoblasts	None	Trophoblast plugging could reduce predicted shear stress in the upstream spiral artery to < 2dyne/cm2, with a corresponding increase in radial artery shear stresses A cohesive trophoblast plug limited predicted blood flow to the intervillous space, but as plug porosity increased the radial arteries became more important modulators of predicted intervillous blood flows
Zamir et al. 2018 [41]	Maternal spiral artery	To create a 3D mathematical model of a helical spiral artery	None	Both vessel length and degree of coiling had effects on spiral artery resistance to blood flow, whilst coiling contributed to turbulent flow patterns
Saghian et al. 2019 [42]	Maternal spiral artery	To develop an agent-based model of trophoblast migration within a plugged spiral artery	None	Predicted trophoblast plug integrity depended upon cell–cell interactions, chemotactic forces and blood flow rates
Zamir et al. 2021 [43]	Maternal spiral artery	To create a 3D mathematical model of spiral artery transformation (reducing in artery coiling, funnelling of distal portion of artery)	None	The extent of spiral artery uncoiling, rather than funnelling, was the more important variable affecting vessel resistance
Cotter et al. 2014 [44]	Interface of maternal circulation with placental villous tree	To develop a stochastic model of villous tree development in early pregnancy, assuming placental villous development is chemotactically driven by factor(s) released from maternal spiral arteries	None	Placental shapes generated by the model mimicked normal biological variation seen in human placentas A disrupted initial distribution of maternal spiral arteries produced model placentas which were less circular and had more peripheral cord insertions
Lecarpentier et al. 2016 [45]	Interface of maternal circulation with placental villous tree	To use 3D models of the intervillous space to simulate syncytiotrophoblast shear stresses in the third trimester	*n* = 1 third-trimester placental specimen from healthy pregnancy *n* = 5 healthy pregnant participants in third trimester studied with dynamic placental MRI	Predicted shear stresses on the syncytiotrophoblast were spatially heterogenous and have lower values than are reported in the rest of the cardiovascular system
Saghian et al. 2017 [46]	Interface of maternal circulation with placental villous tree	To develop a 3D model of blood flow from spiral arteries into the intervillous space throughout gestation	None	Blood flow “jets”, as observed by Doppler ultrasound, could be reproduced by the model The properties of blood flow “jets” were altered depending on the density and organisation of placental villi, particularly after 12 weeks’ gestation
Roth et al. 2017 [47]	Interface of maternal circulation with placental villous tree	To develop a 3D model of blood flow from a spiral artery to the intervillous space in the third trimester	*n* = 3 placental specimens - 1 fetal growth restriction and pre-eclampsia - 1 fetal growth restriction only - 1 uncomplicated pregnancy	Model simulations of pathological conditions predicted the presence of high velocity jets, blood flow vortices, elevated shear stress levels and higher overall pressures in intervillous space (not observed in clinically normal case)
Perazzolo et al. 2017 [48]	Interface of maternal circulation with placental villous tree	To use 3D models of the intervillous space to simulate the effect of maternal blood flow rate on placental solute transfer	Healthy term placental specimens (number not specified)	For physiological ranges, maternal blood flow rate was the main determinant of predicted placental solute transfer
Mekler et al. 2022 [49]	Interface of maternal circulation with placental villous tree	To use a 3D model of the intervillous space to assess the effect of placental villi and decidual vein distribution on placental oxygen transfer	None	Larger villi-free regions at spiral artery inlets, and more peripheral and asymmetric distributions of decidual veins, were predicted to improve placental oxygen uptake
Lee et al. 2023 [50]	Interface of maternal circulation with placental villous tree	To develop a 3D model of blood flows within the intervillous space in the first and third trimesters	*n* = 6 placental specimens - 3 first-trimester placentas - 3 term placentas	Intervillous spaces at term had lower predicted shear stresses and pressure gradients than first trimester intervillous spaces
Najmi et al. 2024 [51]	Interface of maternal circulation with placental villous tree	To use a 3D model of the intervillous space to assess the effect of placental villi distribution, and the anatomy of spiral artery and decidual veins, on maternal blood flow	None	Extent of spiral artery remodelling, decidual vein location and diameter, and cavity length and diameter had significant effects on predicted intervillous blood flow patterns Partial spiral artery remodelling and wide villi-free cavities (as are associated with clinical placental disease) induced areas of flow recirculation in models, potentially corresponding to reduced placental efficiency
Hutchinson et al. 2025 [52]	Interface of maternal circulation with placental villous tree	To use a model of maternal blood flow in the intervillous space to investigate and explain observed features from placental diffusion-weighted MRI	MRI dataset gathered from *n* = 22 pregnant participants in the third trimester - 9 with pregnancy complications (pre-eclampsia or fetal growth restriction) - 13 with uncomplicated pregnancies Comparison of individual MRI datasets informed interpretation of simulated results but were not used directly to personalise models	Model simulations suggested that complex blood flow patterns adjacent to spiral artery inlets and venous outlets were responsible for observed MRI features close to the maternal wall and placental periphery

#### Computational approaches to the pregnant maternal circulation

One-dimensional computational fluid dynamics models predict flow and wave propagation phenomena by simulating blood flow through a cardiovascular network as analogous to current running through an electric circuit. So-called “open loop” models consider an isolated segment of the network (typically on the arterial side) with a prescribed inflow and “free” outflow into microvascular beds. In contrast, “closed loop” models comprise both arterial and venous segments, with blood pumped around the network in a circular manner.

Corsini et al. first proposed a closed-loop model of the pregnant maternal circulation, which reproduced realistic trends in haemodynamic variables and flow patterns when tested with literature-reported cardiovascular parameters for each trimester of normal pregnancy [25]. This model included a simplified uteroplacental blood supply arising exclusively from the uterine arteries, and was validated with population-based parameters.

This closed-loop circulatory model of pregnancy was further developed by Carson et al., to incorporate a more comprehensive uteroplacental circulation, including utero-ovarian communicating arteries, arcuate and downstream radial or spiral arteries, and the placental vascular bed [26]. Their model simulations, using data from two healthy participants, produced realistic estimates of uterine blood flow and uterine artery waveforms in line with literature-reported metrics, for each trimester of pregnancy and the postpartum period.

In a follow-up study, the authors parameterised the same model using second trimester cardiovascular measurements from two women with early-onset fetal growth restriction and pre-eclampsia, and two with chronic hypertension but no placental disease [27]. Uterine artery waveforms produced in model simulations were similar to those measured by Doppler ultrasound in each patient, and included the presence of early diastolic notching (a clinically significant radiographic feature indicating increased placental resistance) for those with pre-eclampsia.

In addition to predictions of blood flow and arterial waveforms, computational circulatory models can also generate estimations of vessel diameters. Carson et al. found the main systemic arteries to have higher model-predicted diameters in their pre-eclamptic subjects, in line with previous clinical reports which suggest increased aortic diameter may also persist following delivery for women with pre-eclampsia [27, 61, 62]. Predicted spiral artery diameters did not show a consistent trend between the groups.

The above closed-loop circulatory models simulate blood volume increase and redistribution between vascular compartments during pregnancy by incrementally adjusting blood volume in the venous compartment, to achieve the subject’s observed cardiac output [26, 27]. However, only measured data from systemic arteries was used for model parameterisation, with healthy pressures based on literature in non-pregnant subjects assumed for pulmonary and systemic venous circulations. The effect of pregnancy-related changes in venous haemodynamics on cardiac preload, as well as the role of increasing intra-abdominal pressure secondary to a gravid uterus, were not considered [63, 64].

Incorporating these often-overlooked aspects of gestational physiology in future model design and parameterisation could generate opportunities to better understand the role of venous function in healthy and disordered pregnancies. Indeed, recent work has attempted to use circulatory models to simulate the effect of inferior vena cava compression by the uterus on maternal haemodynamics in the prone position [30].

These preliminary studies are also clearly limited by extremely small cohort sizes. Further validation and application of the models must involve larger patient datasets, particularly when considering the phenotypically heterogenous disease of pre-eclampsia. Recent and ongoing research efforts are using one-dimensional circulatory models to predict downstream flows and pressures in small uteroplacental vessels, which cannot feasibly be measured directly [31]. These may indicate abnormal patterns of vascular adaptation prior to overt clinical manifestations of pre-eclampsia or fetal growth restriction with greater accuracy than currently available biomarkers.

#### An algorithm for cardiac remodelling

Increases in circulating fluid volume and cardiac output during pregnancy are accompanied by structural changes in the heart muscle itself. Atrial and ventricular end-diastolic volumes and cardiac wall mass all expand throughout gestation and may not revert to baseline until at least a year postpartum [65, 66]. There is evidence of exaggerated or abnormal cardiac remodelling during pre-eclamptic pregnancies, with greater degrees of left ventricular remodelling and echocardiographic signs of diastolic dysfunction which may precede the onset of clinical disease [67, 68].

Applying bioengineering principles to pregnancy-related cardiac remodelling could help elucidate the mechanisms which underlie this phenomenon, and which become disordered in pregnancy disease. By implementing a cardiac remodelling algorithm within a closed-loop circulatory model of the maternal circulation, Comunale et al. demonstrated that cardiac growth during pregnancy could be driven by haemodynamic stimuli when constrained by a requirement to maintain homeostasis of cardiac myofiber stress and chamber wall shear stress [32].

This model is yet to be validated with individual patient-level data and does not account for potential influences from hormonal signalling or drugs on cardiac muscle. Nevertheless, such approaches could pave the way for the development of multiscale models which incorporate cell-level systems biology with mechanic and haemodynamic approaches to determine the factors which drive abnormal cardiac remodelling in pre-eclampsia [60].

A strength of this model for studying cardiac physiology in pregnancy is its use of female-specific haemodynamic parameters for model calibration [69]. However, in recent years our understanding of sex-specific differences in cardiovascular phenotypes has dramatically expanded, with an appreciation that women exhibit different myocardial and vascular responses to stress and ageing compared with men [70]. Computational models simulating cardiac and circulatory changes in pregnancy may therefore need to revise their baseline assumptions based on historical clinical observations of male physiology, in light of this emerging evidence [71].

#### Models of uterine vascular adaptation

Dramatic growth and remodelling of the local uterine vasculature is vital for a successful pregnancy outcome, and may be impaired in pre-eclamptic or growth-restricted pregnancies [72, 73]. The resolution of ultrasound and MRI imaging limits current attempts to study dynamic structural changes of arcuate and radial vessels within the uterine muscle in vivo. Incorporation of blood vessel growth into cardiovascular models, as proposed by Gleason et al., could therefore provide novel insights into this elusive part of the maternal circulation [28].

Computational modelling which takes account of maternal vascular growth and remodelling can reproduce haemodynamic trends observed in early and late forms of pre-eclampsia, including the increased stiffness of large systemic arteries seen at early gestations [29, 74]. However, these simulations have been parameterised using population-level haemodynamic metrics from literature, drawn from published studies with wide variation in their definitions of early and late pre-eclampsia. Robust validation of these models requires more comprehensive datasets of haemodynamic metrics, linked to pregnancy outcomes, from individuals with uncomplicated and disordered pregnancies.

In contrast to the closed-loop network models that capture the pressure and flow changes across the wider maternal circulation, open-loop models have been employed to study the pregnant uterine vasculature in isolation. These have allowed researchers to interrogate the contribution of individual components of the remodelled uterine vasculature to the uterine artery waveform, and to create computationally expensive three-dimensional reconstructions of uterine arterial networks which account for the effects of changing bifurcation angles and vessel tortuosity with advancing gestations [34, 37]. These three-dimensional simulations also provide a means of predicting haemodynamic metrics, such as wall shear stresses, which are difficult to measure in uterine vessels in vivo [39]. Whilst there have been attempts to use measures of blood velocities from 4D flow MRI to personalise uterine vascular models to individual pregnant subjects, the constraint of imaging resolution has limited this approach to larger (common iliac, external iliac and uterine) pelvic vessels [36].

Computational models of the remodelled pelvic vasculature have been employed to assess a clinical intervention, specifically internal iliac balloon occlusion as a treatment to prevent major obstetric haemorrhage at caesarean section for pernicious placenta praevia [35]. When applied to a retrospective analysis of clinical cases, a modelling approach demonstrated the importance of collateral circulation in explaining observed blood losses at delivery. The profoundly abnormal uteroplacental anatomy seen in pernicious placenta praevia may limit the applicability of this work to pregnancies in other contexts [75]. Nevertheless, this is a compelling example of the utility of computational modelling as a tool to understand, and potentially predict, clinical observations in pregnancy disease.

#### Mechanistic insights into spiral artery remodelling

Progressive transformation of spiral arteries within the uterine endometrium, from tightly coiled, highly muscularised vessels to wide non-muscular conduits, is critical for maintaining placental blood supply as gestation advances [7, 76]. This dramatic remodelling is associated with spiral artery colonisation with extravillous trophoblasts, which migrate into the decidua from placental anchoring villi during the first trimester [77]. During this process, invading extravillous trophoblasts form “plugs” within spiral artery lumens, which may persist until mid-gestation [72]. The physiologically hypoxic downstream environment created by this partial occlusion to blood flow is thought to be critical for early placental development [78, 79].

Defective spiral artery transformation is seen in placental bed biopsies from pre-eclamptic and growth restricted pregnancies, but cannot be feasibly detected in vivo [80]. Computational modelling provides opportunities to interrogate this process, for example by demonstrating the effects of altered spiral artery morphology and deficient trophoblast plugging on upstream and downstream blood flows [38, 40, 41, 43]. In silico models also provide a means of investigating the chemotactic and haemodynamic factors which influence trophoblast migration and plug disintegration, and thus advance understanding of the cellular mechanisms underlying normal and disordered development of the uteroplacental vasculature [42].

#### Modelling interactions at the maternal–fetal interface

Maternal spiral arteries open out into the placental intervillous space, an environment with low resistance to blood flow which forms the interface between maternal blood and the fetal villous tree. Progressive development and branching of fetal villi expand the capacity for oxygen and nutrient exchange between maternal and fetal circulations, and are impaired in growth restricted pregnancies [81]. Placental villous development and maternal uterine vascular remodelling occur in tandem, with each process exerting paracrine and haemodynamic influences on the other.

The potential for chemotactic agents from maternal spiral arteries to mediate villous tree branching has been demonstrated with computational simulations, which give rise to realistic two-dimensional placental shapes [44]. These models generate placental shapes which are less circular and have more peripheral cord insertions under starting conditions that mimic poorly developed networks of maternal spiral arteries, suggesting a possible mechanistic link between abnormal maternal vascular adaptation and the abnormal placental morphology observed in fetal growth restriction [82].

Expression of mechanosensing proteins by the syncytiotrophoblast begins in the first trimester, suggesting that shear stresses from maternal blood flow in the intervillous space may have an important effect on placental villous development [50]. Computational modelling studies have demonstrated that these shear stresses are highly spatially heterogenous and vary substantially throughout gestation [45, 50]. Detailed fluid dynamic simulations can reproduce complex patterns of blood flow, including the “jets” observed at spiral artery openings in Doppler ultrasound studies, and relate these to villous tree and decidual vein anatomies [46, 49, 51, 83].

Modelling blood flows at the placental interface with this level of resolution involves reconstructing the intervillous space in three dimensions, using detailed histology or imaging of ex vivo placental tissue. Reconstructions generated from pre-eclamptic or growth restricted placentas display marked differences compared with those based on healthy specimens, including higher levels of wall shear stresses at the villous tree surface and the formation of blood flow vortices within the intervillous space [47].

Detecting pathological patterns of intervillous blood flow and placental morphogenesis as they develop remains a persisting challenge for researchers and clinicians. Recent advances in dynamic MRI and contrast-enhanced ultrasound hold promise for enabling assessment of in vivo placental blood flows if safety concerns can be resolved [84, 85]. Indeed, recent work by Hutchinson et al. used a computational model of maternal intervillous circulation to link MRI signal features to functional blood flow parameters [52]. Harnessing advanced imaging modalities to generate personalised models of blood flow and solute transfer at the placental interface would be an important step towards linking individual anatomy to function, and help demystify the “black box” of placental development during pregnancy disease [48, 49].

## Discussion

### Personalised cardiovascular models in the “digital twin” era

Cardiovascular adaptation to pregnancy is clearly a dynamic, complex process with profound implications for maternal and fetal health. Combining increasingly sophisticated modelling techniques, such as those described in this review, with data analytics and artificial intelligence technologies may one day enable the creation of highly detailed and precise virtual representations of the evolving maternal physiology throughout gestation [86, 87]. These “digital twins” could represent valuable tools for clinicians by improving prediction of adverse outcomes and guiding more personalised, proactive intervention in high-risk and complicated pregnancies.

Such a future will require the integration of computational models across diverse maternal organ systems including cardiac, renal, cerebral, and uterine circulations [33, 60, 88]. Maternal digital twins should also incorporate established and novel measures of endothelial dysfunction and angiogenesis, given the association of pregnancy disorders with widespread microcirculatory changes and the proven potential of angiogenic biomarkers Placental Growth Factor and soluble fms-like tyrosine kinase-1 for pre-eclampsia prediction and diagnosis [89–94].

High quality data will be vital to create accurate personalised virtual representations of pregnant individuals. Novel functional ultrasound and MRI imaging-based techniques are allowing increasingly sensitive and detailed assessments of haemodynamics throughout the maternal circulation, including at the level of blood flow and solute flux at the placental interface [84, 95–98]. Meanwhile modern wearable technologies could allow digital twins to continuously update with real-time cardiovascular data, mirroring the evolution of maternal physiology on a day-to-day, hour-by-hour and minute-by-minute basis [99]. Robust assessment and quantification of the effects that model input variability and uncertainty have on the behaviours and predictive outputs of model will be essential for their safe, effective clinical translation (as has been recognised in the field of cardiovascular physiology more widely) [100].

Data used to validate computational models of pregnancy must also be representative of diverse populations. Stark ethnic disparities have been reported in the occurrence, presentation, and outcomes of pre-eclampsia and fetal growth restriction [101, 102]. However, only two of the modelling studies in this review which utilised data from pregnant women or clinical samples reported on the ethnicity of participants [30, 31]. Systematic reporting of ethnicity in future modelling studies will be essential to ensure appropriate representation of minority groups and tackle health disparities.

To be genuinely predictive of adverse obstetric outcomes, a digital twin of pregnancy must integrate models of maternal, placental, and fetal circulations and reflect the continuous interactions between these intimately connected systems. Computational approaches to the study of placental and fetal haemodynamics in isolation are developing apace, but must overcome substantial and unique challenges of their own.

For example, computational models of blood flows within the placental villous tree depend on anatomical information determined from ex vivo specimens, which are inevitably affected by post-delivery processing, and must account for the haemodynamic implications of blood’s particulate nature at the microscopic capillary scale [103, 104]. The fetal circulation is difficult to interrogate with imaging (in no small part due to artefacts arising from unpredictable fetal movements), but modelling has shown promise in simulating fetal blood flow redistribution in the context of growth restricted pregnancy, amongst other pathologies [105]. New insights into fetal vascular responses to the intrauterine and extrauterine environments, generated by advancing fetal MRI methodologies, could hugely expand the level of anatomical and functional detail of future models [106].

### Translating cardiovascular models from the computer to the clinic

The current clinical paradigm for diagnosing and managing dysfunctional cardiovascular responses to pregnancy is inadequate. Our strategies to identify pathologically compromised fetuses, and distinguish them from their constitutionally small but healthy peers, are based on the use of population-based growth charts of uterine size and ultrasound biometry, combined with markers of late-stage compensation in the form of fetal blood flow redistribution and abnormal heartrate patterns. This strategy fails to detect a significant proportion of growth restricted babies, too often with tragic consequences [107].

Even in pregnancies where vascular and placental dysfunction are identified, clinicians have no tools which can accurately forecast disease trajectories or times to deterioration. A framework which can guide timely delivery in pre-eclamptic and growth restricted pregnancies, and avoid the harms associated with unnecessary iatrogenic prematurity, is sorely needed [108, 109].

Computational modelling has the potential to connect measurable macrovascular phenomena to physiological processes throughout maternal, placental, and fetal circulations. Validation, verification, and uncertainty quantification remain challenges, particularly for complex models [110]. However, if these can be overcome, multiscale computational approaches could unlock deeper systems-level understanding, and inform more nuanced management, of cardiovascular maladaptation to pregnancy.

To realise the promise of this emerging field to improve maternal and fetal outcomes, models should be developed in line with agreed reproducibility and credibility standards, and validated in large, diverse pregnancy cohorts across a wide range of gestations [111]. Personalised simulations should utilise data which is feasible to collect, scalable, and acceptable to pregnant women, and produce clinically meaningful outputs suitable for integration into clinical workflows [112]. Computational pipelines must be streamlined so that model outputs can be generated rapidly enough to guide management, and uncertainties associated with model predictions must be robustly defined [113]. The potential of artificial intelligence to reduce processing time for running computational models, boost the power of clinical modelling studies, and identify obscure patterns associated with disease, should be balanced against the need for large training datasets, data privacy concerns, poor explainability, and potential for algorithm bias, over-fitting, and incorrect predictions [114].

Finally, ensuring both the applicability and accessibility of this fledgling technology for disadvantaged populations must be a priority, given these groups bear a disproportionate burden of obstetric morbidity and mortality [115, 116].

### Strengths and limitations

In this scoping review, we have employed a registered, pre-specified protocol, and a systematic, reliable process using multiple databases to identify relevant studies from the last twelve years. Study selection criteria and data extraction were reliably applied by two researchers. We used an inclusive approach to identify as much relevant data as possible.

Despite this, we may not have identified all relevant studies; in particular, our database search may have been limited by poorly indexed literature in the emerging and interdisciplinary field of computational modelling in pregnancy. We made efforts to mitigate this limitation by reviewing references of retained articles and through discussion with experts, to identify studies not captured in the search.

## Conclusion

This scoping review sheds light on how computational modelling has been applied to different aspects of the pregnant maternal cardiovascular system. These approaches could reveal deeper insights into healthy and disordered pregnancies than are afforded by current research methodologies, and identify novel strategies to guide risk stratification, monitoring, and timely intervention. Validation in large, representative pregnancy cohorts, inclusion of clinically accessible and scalable metrics for model personalisation, and concurrent development of rapid and robust computational processing pipelines, will be vital if these tools are to be harnessed for the provision of personalised obstetric care.

## Supplementary Information

Supplementary Material 1.

## Data Availability

All data generated or analysed during this study are included in this published article and its supplementary information files.
