# Supplementary material for: Modelling maternal cardiovascular adaptation to pregnancy: a scoping review
Source: BMC Pregnancy Childbirth. 2025 Nov 24;26:88. doi: 10.1186/s12884-025-08270-z (PMC12838005; doi:10.1186/s12884-025-08270-z)
Supplement: Supplementary file 1 — Supplementary Material 1. [file 12884_2025_8270_MOESM1_ESM.docx]

Appendix S1: Search Strategy

**OVID MEDLINE**

<January 01, 2013 to September 01 2025>

1. pregnancy/ or maternal-fetal exchange/ or placentation/ or pregnancy, high-risk/

2. pregnancy complications/ or fetal death/ or stillbirth/ or fetal diseases/ or fetal growth retardation/ or hypertension, pregnancy-induced/ or eclampsia/ or hellp syndrome/ or pre-eclampsia/ or placenta diseases/ or abruptio placentae/ or placental insufficiency/ or pregnancy complications, cardiovascular/

3. 1 or 2

4. cardiovascular system/ or blood vessels/ or arteries/ or umbilical arteries/ or uterine artery/ or veins/ or heart/ or fetal heart/ or ductus arteriosus/ or truncus arteriosus/ or heart atria/ or heart conduction system/ or atrioventricular node/ or "bundle of his"/ or purkinje fibers/ or sinoatrial node/ or heart septum/ or heart valves/ or heart ventricles/ or myocardium/

5. blood circulation/ or placental circulation/ or regional blood flow/

6. blood flow velocity/ or cardiac output/ or stroke volume/ or hemodynamic monitoring/ or pulse wave analysis/

7. blood pressure/ or heart rate/

8. umbilical cord/ or umbilical arteries/ or umbilical veins/

9. atrial remodeling/ or vascular remodeling/ or ventricular remodeling/

10. cardiomegaly/ or hypertrophy, left ventricular/ or hypertrophy, right ventricular/ or heart failure/ or heart failure, diastolic/ or heart failure, systolic/

11. placenta/ or chorionic villi/ or decidua/ or trophoblasts/

12. 4 or 5 or 6 or 7 or 8 or 9 or 10 or 11

13. (((computation* or digital or mathematic* or network or haemodynamic* or hemodynamic* or cardiovascular or vascular or circulat* or data-driven or multiscale or multi-scale or flow or wave or fluid or personal* or patient-specific or "patient specific" or "in silico") adj3 model*) or digital twin).mp. [mp=title, abstract, heading word, drug trade name, original title, device manufacturer, drug manufacturer, device trade name, keyword heading word, floating subheading word, candidate term word]

14. 3 and 12 and 13

**OVID EMBASE**

<January 01, 2013 to September 01 2025>

1. pregnancy/

2. maternal hypertension/ or pregnancy complication/ or hellp syndrome/

3. preeclampsia/ or "eclampsia and preeclampsia"/

4. stillbirth/ or fetus death/

5. placenta disorder/ or placenta insufficiency/

6. pregnancy disorder/ or high risk pregnancy/

7. fetus disease/ or intrauterine growth retardation/

8. intrauterine growth retardation/ or selective intrauterine growth restriction/ or small for gestational age/

9. pregnant woman/

10. gestational age/

11. gestation period/

12. perinatal period/

13. placenta/ or fetoplacental unit/

14. 1 or 2 or 3 or 4 or 5 or 6 or 7 or 8 or 9 or 10 or 11 or 12 or 13

15. cardiovascular system/ or blood vessel/ or heart/ or systemic circulation/

16. blood vessel/ or peripheral vascular system/

17. systemic circulation/ or placenta circulation/

18. heart/ or fetus heart/ or heart atrium/ or heart septum/ or heart valve/ or heart ventricle/

19. heart ventricle muscle/

20. heart ventricle remodeling/

21. blood pressure/ or artery perfusion pressure/ or blood pressure fluctuation/ or blood pressure variability/ or diastolic blood pressure/ or heart atrium pressure/ or heart ventricular pressure/ or pulse pressure/ or systolic blood pressure/ or venous pressure/

22. heart output/

23. circulation/ or arterial circulation/ or fetus circulation/ or organ circulation/ or peripheral circulation/ or venous circulation/ or venous return/

24. cardiovascular function/ or blood vessel function/ or cardiovascular performance/ or cardiovascular response/ or heart function/ or hemodynamics/

25. hemodynamics/ or blood flow/ or blood pressure/ or blood rheology/ or blood vessel capacitance/ or cardiopulmonary hemodynamics/ or cardiovascular autoregulation/ or heart hemodynamics/ or hemodynamic stress/

26. blood flow/ or artery blood flow/ or capillary flow/ or "organ and tissue blood flow"/ or vein blood flow/

27. pulse wave velocity/ or arterial stiffness parameters/ or aortic pulse wave velocity/ or arterial pulse wave velocity/

28. arterial stiffness/

29. arterial stiffness parameters/ or augmentation index/

30. uterine artery/

31. umbilical cord/ or umbilical artery/ or umbilical cord blood/ or umbilical vein/

32. placenta/ or chorioallantois/ or decidua/ or fetoplacental unit/ or placenta circulation/

33. doppler flowmetry/ or flow measurement/

34. artery/

35. vein/

36. artery blood flow/

37. blood flow velocity/ or hemodynamic parameters/

38. 15 or 16 or 17 or 18 or 19 or 20 or 21 or 22 or 23 or 24 or 25 or 26 or 27 or 28 or 29 or 30 or 31 or 32 or 33 or 34 or 35 or 36 or 37

39. (((computation* or digital or mathematic* or network or haemodynamic* or hemodynamic* or cardiovascular or vascular or circulat* or data-driven or multiscale or multi-scale or flow or wave or fluid or personal* or patient-specific or "patient specific" or "in silico") adj3 model*) or digital twin).mp. [mp=title, abstract, heading word, drug trade name, original title, device manufacturer, drug manufacturer, device trade name, keyword heading word, floating subheading word, candidate term word]

40. 14 and 38 and 39

**WEB OF SCIENCE CORE COLLECTION**

<January 01, 2013 to September 01 2025>

(computation* OR digital OR mathematic* OR personal* OR haemodynamic* OR hemodynamic* OR cardiovascular OR vascular OR data-driven OR multiscale OR multi-scale OR circulatory OR fluid OR flow OR network OR wave OR "in silico" OR "patient-specific") NEAR/3 model* (Topic) and pregnancy OR gestation OR placenta* OR pre-eclampsia OR preeclampsia OR stillbirth OR "fetal growth restriction" (Topic) and cardiovascular OR vascular OR arter* OR vein* OR "umbilical cord" OR circulation OR "blood flow" OR "pulse wave" OR heart OR placenta OR "blood supply" (Topic)
